# Inter-individual variation in adaptive capacity at onset of lactation: Linking metabolic phenotype with mitochondrial DNA haplotype in Holstein dairy cows

**DOI:** 10.1038/s41598-018-33853-6

**Published:** 2018-10-18

**Authors:** Asako Kinoshita, Ákos Kenéz, Martin Hasselmann, Sven Dänicke, Korinna Huber

**Affiliations:** 1 Institute of Animal Science, Faculty of Agricultural Sciences, University of Hohenheim, Stuttgart, 70599 Germany; 2 Institute of Animal Nutrition, Friedrich-Loeffler-Institute, Braunschweig, 38116 Germany; 3 Present Address: College of Veterinary Medicine and Life Sciences, City University of Hong Kong, Kowloon, Hong Kong SAR

## Abstract

Modern-day dairy cows express great variation in metabolic capacity to adapt to the onset of lactation. Although breeding programs increased the breeding value for longevity and robustness in the sires, a respective phenotype in female offspring has not been improving as predicted. Fundamental energy generating pathways such as mitochondrial fatty acid oxidation might have a crucial role for robustness and metabolic efficiency in dairy cows. Therefore, mitochondrial enzyme expression was examined in liver samples of one set of animals before and after calving. Furthermore, the mitochondrial DNA sequence was determined for each individual of a second set of animals using liver samples. Results from the first trial indicate that the expression and extent of phosphorylation of acetyl-CoA carboxylase (ACC) is the major key step for modulating fatty acid flux into the mitochondria at early onset of lactation in Holstein dairy cows. In the second trial, mitochondrial DNA sequencing and identification of mutation patterns yielded three major haplotypes. Haplotype H2 was closely associated with liver fat content, plasma glycerol and acyl-carnitine concentrations. The mitochondrial DNA haplotype, which is a feature of the maternal lines, might be related to the inter-individual variation in metabolic capacity of Holstein dairy cows.

## Introduction

A highly tensed metabolic condition is triggered at the onset of lactation by the great lactation performance in cows of modern-day intensive dairy production systems. Cellular energy metabolism by oxidative phosphorylation (OXPHOS) and ATP production in the mitochondrial respiratory chain is thereby the key event to provide sufficient fuel for synthesis of milk components and for maintenance of vital physiological functions of the cow. At onset of lactation, feed intake becomes reduced in dairy cows due to endocrine changes around parturition and parturition stress; thus, low energy intake in combination with high energy outflow via milk causes a negative energy balance (NEB). The animal responds to the NEB by lipid mobilization releasing non-esterified long- and medium chain fatty acids (NEFA) into the blood stream. The liver mitochondria are the major place where utilization of NEFA by beta-oxidation and OXPHOS (both together are referred to as fatty acid oxidation, FAO) takes place. However, a high inter-individual variation in the capacity to adapt to onset of lactation was observed in dairy cows, particularly within the high-yielding Holstein breed.

Limitations of FAO in Holstein dairy cows were recently reviewed^1^. Ketosis and liver steatosis are potential consequences of hepatic catabolic failure; and most likely also metabolic inefficiency in clinically healthy individuals might be based on variations in mitochondrial functionality^2^. The first step of mitochondrial NEFA utilization is the activation of fatty acids by esterification to CoA and then formation of acyl-carnitines from acyl-CoA by carnitine palmitoyltransferase (CPT1)^3^, a key pathway which was also described for ruminants. Liver CPT1 activity and sensitivity to malonyl-CoA, its metabolic regulator, were not affected by prepartum energy intake or postpartum health status^4^. In muscle of dairy cows, an upregulation of CPT1 mRNA in early lactation was observed^5^. CPT1 is discussed as rate limiting step to transport acyl-carnitines across the outer mitochondrial membrane. Transport into the mitochondrial matrix is then performed by carnitine/acyl-carnitine translocase (CACT) located in the inner mitochondrial membrane. In the mitochondrial matrix, carnitine is released by CPT2 and acyls are re-esterified to CoA to be degraded in beta-oxidation to acetyl-CoA. Acetyl-CoA is channelled into the tricarboxylic acid cycle which provides reduction equivalents for OXPHOS and ATP generation. If there are limitations to introduce acetyl-CoA to the cycle, ketone body production increases. This pathway is mediated by a hepatic mitochondria-specific enzyme, the ketogenic 3-hydroxy-3-methylglutaryl-coenzyme A synthase 2 (HMGCS2), synthesizing acetoacetyl-CoA. Messenger RNA abundance and protein content of HMGCS was reduced in the liver of ketotic cows possibly indicating a feedback inhibition of expression by increased ketone body availability^6^. Acyl-CoA produced by Acyl-CoA synthase long chain (ACSL1), which is not used for acyl-carnitine shuttling via CPT1, CACT and CPT2, could also be re-esterified to triacylglycerol and deposited in the liver cells causing hepatic steatosis. This enzyme was significantly upregulated in ketotic cows^6^.

Once NEFA are channelled through the carnitine shuttle system and were degraded in beta-oxidation to acetyl-CoA, ATP generation depends on the capacity of the respiratory chain in the mitochondrial matrix. OXPHOS function of mitochondria is determined by genes which are encoded in the nuclear genome but also by genes encoded in the mitochondrial genome, the mitochondrial (mt)DNA. This small mtDNA double ring is 16.3 kB in size and consists of 37 genes coding for 22 tRNA, for 2 rRNA and for 13 subunits of enzymes of the respiratory chain without any introns^7^. The non-coding displacement (D) loop region was used for many approaches to assess ancestral relationships between cattle breeds (e.g. *Bos taurus* versus *Bos indicus)* by defining their maternal lineages^8^. From the evolutionary perspective, mitochondria originated from invaded symbiotic bacteria during evolution and co-evolved with the host, since many of the mitochondrial proteins are encoded by nuclear genes (reviewed)^9^. Consequently, the close interaction between host nuclei and mitochondria, requires a high compatibility between both genomes for a proper mitochondrial functionality; mismatches between both genomes could cause severe perturbations of OXPHOS capacity^10^. There is scientific evidence since decades that there is a close relationship between mtDNA haplotypes and health, long lifespan, performance and fertility in human and farm animals. In general, mitochondria are dynamically involved in several cell functions besides respiration and ATP energy generation such as cellular aging and death, chronic inflammation and oxidative stress (reviewed)^9^. Studies on mtDNA haplotypes showed a close relationship with certain mutations in Japanese centenarians and support the idea that lower susceptibility to adult-onset of diseases is based on a mtDNA haplotype predisposing resistance^11^. In a Holstein dairy cow herd with 36 different maternal lineages, significant negative or positive associations between mtDNA haplotype and overall production means in milk yield, milk fat and milk energy were found, indicating that mitochondrial genome is modulating energy metabolism^12^. Interestingly, only the sequence of the non-coding region of the mtDNA, the D loop was used in that study. In beef cattle, a significant association between mtDNA haplotypes (based on D loop and ND-5 gene) and calving rate was found^13^. However, a more detailed description of the metabolic phenotype related to distinct mtDNA haplotypes is lacking in farm animals. Furthermore, determination of the individual mtDNA haplotype in Holstein cows to understand inter-individual variation in metabolic efficiency was not examined so far.

Therefore, aims of this study were to identify linked conditions in metabolic phenotype and mitochondrial genotype to potentially explain the high inter-individual variation in dairy cows’ capacity to adapt to the onset of lactation. This was targeted by (1) describing the molecular changes of mitochondrial energy metabolism, particularly with respect to the carnitine/acyl-carnitine shuttle system across mitochondrial membranes in 21 Holstein dairy cows throughout the periparturient period (trial 1); and (2) determining the sequences of mtDNA of 26 Holstein dairy cows and to identify mtDNA haplotypes and matching conventional hepatic phenotype characteristics to identified mtDNA haplotypes (trial 2).

Samples of trial 1 and 2 were acquired in a larger experimental approach from Holstein dairy cows. Results of that approach were already published earlier^2,14–17^. The metabolomics analysis performed in these cows came to the conclusion that mitochondrial function might be essential for proper metabolic adaptation to onset of lactation^2^. Thus, the same samples were subjected to further processing and analysis to assess potential pathways involved in individual mitochondrial functionality.

## Results and Discussion

### Mitochondrial energy metabolism throughout the periparturient period

**Figure 1:**
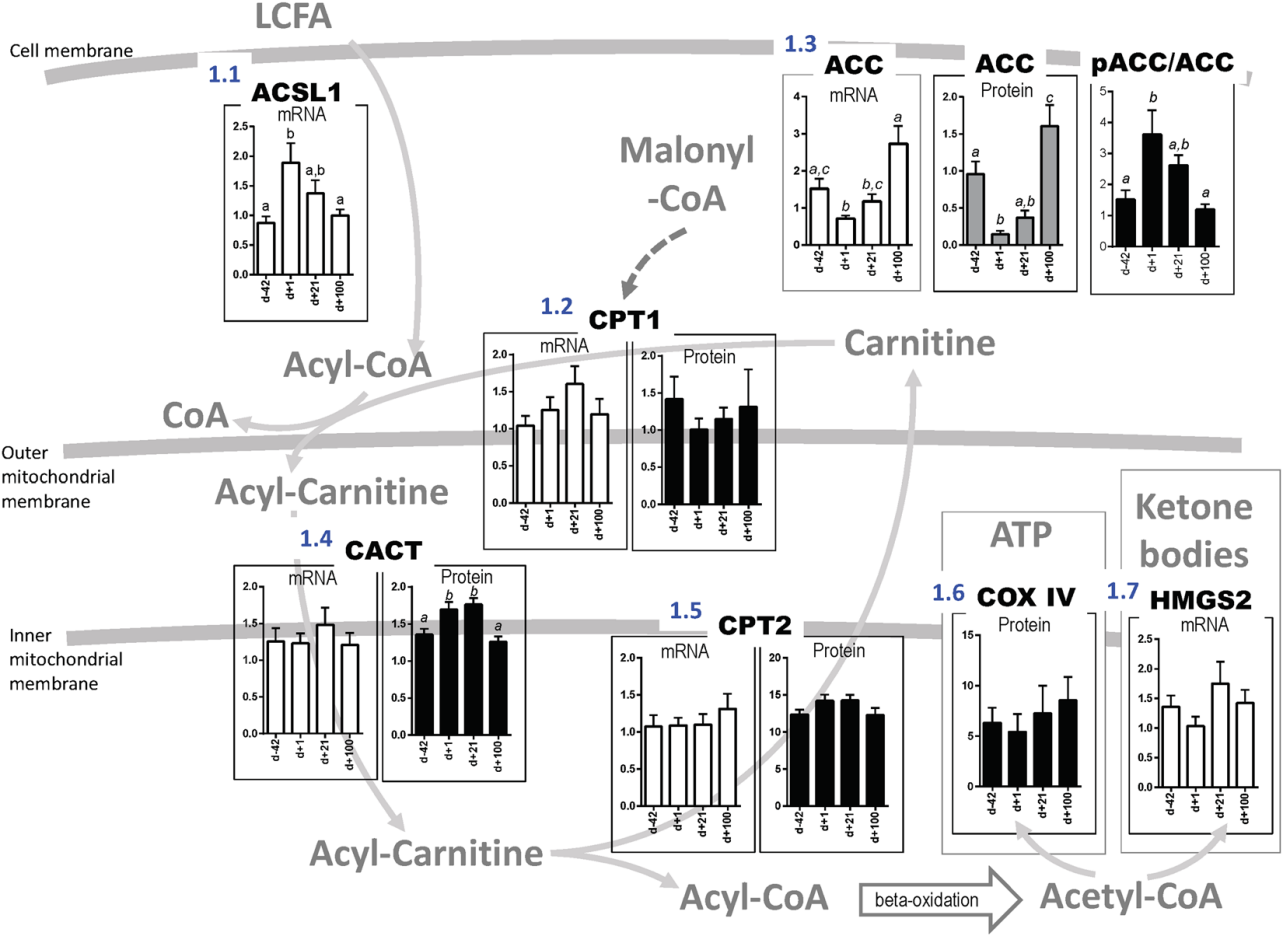
Mitochondrial energy metabolism throughout the periparturient period. Shown are relative amounts of mRNA and protein and extent of phosphorylation, respectively, of key enzymes involved in carnitine/acyl-carnitine shuttle system (ACSL1: Acyl-CoA synthetase long-chain family member 1; ACC: acetyl-CoA carboxylase; pACC: phosphorylated acetyl-CoA carboxylase (at inhibitory site serine 79); CPT1: carnitine palmitoyltransferase 1; CACT: carnitine-acylcarnitine translocase; CPT2: carnitine palmitoyltransferase 2), respiratory chain (COX IV: cytochrome c oxidase subunit 4) and ketogenesis (HMGS2: 3-hydroxy-3-methylglutaryl-Coenzyme A synthase 2). Bar charts demonstrate expression and extent of phosphorylation at days −42 prepartum, +1, +21 and +100 postpartum in 21 Holstein dairy cows, means ± SEM. One Way Repeated-Measures ANOVA for factor time was performed: (1.1) ACSL1 mRNA p ≤ 0.01; (1.2) CPT1 mRNA, protein n.s.; (1.3) ACC mRNA, protein, phosphorylated protein p ≤ 0.01; (1.4) CACT mRNA n.s., protein p ≤ 0.01; (1.5) CPT2 mRNA n.s., protein p ≤ 0.01; (1.6) COX IV protein n.s.; (1.7) HMGS2 mRNA n.s. Differences between days (1.1–1.7) were assessed by Tukey’s multiple comparison posttest (a,b,c indicate differences with at least p ≤ 0.05).

To describe the molecular pathways of hepatic metabolic adaptation before and after onset of lactation in Holstein dairy cows, the expression of mRNA and proteins for genes related to mitochondrial function in the liver was measured in 21 healthy animals at −42 (d − 42) prepartum and +1, +21, and +100 days (d + 1, d + 21, d + 100) postpartum. Results are demonstrated in Fig. 1. Both protein and mRNA expression levels were quantified for genes related to the hepatic carnitine shuttle (carnitine palmitoyl transferase 1 and 2: CPT1 and CPT2, carnitine/acyl-carnitine translocase: CACT and acetyl-CoA-carboxylase: ACC). Extent of phosphorylation of ACC at serine 79 (inhibitory phosphorylation site) was also determined. Only mRNA expression was quantified for acyl-CoA-synthetase 1 (ACSL1), and hydroxymethylglutaryl-CoA synthase 2 (HMGS2), while only protein expression was quantified for AMP-activated protein kinase (AMPK) and cytochrome c oxidase subunit 4 (COXIV).

Long chain fatty acids (LCFA) are esterified by ACSL1 to form activated acyl-CoA. Compared to d −42 prepartum, ACSL1 mRNA expression was significantly higher at d +1 postpartum but decreased continuously thereafter until d +100 postpartum (Fig. 1.1). If this expression pattern was reflected on protein and functional level, this enzyme was strongly involved in managing the increase of NEFA availability in plasma derived from enhanced lipid mobilization postpartum. Acyl-CoA are processed by CPT1 to form acyl-carnitines and transport them across the outer mitochondrial membrane. Interestingly, neither CPT1 mRNA nor protein expressed a significant adaptive response to onset of lactation (Fig. 1.2). ACC was expressed at the lowest amounts on d +1 postpartum at mRNA and protein level (Fig. 1.3); however, considering protein phosphorylation level, pACC to ACC ratio was significantly higher at d +1 postpartum compared to d −42 prepartum and decreased later again over time postpartum (One Way Repeated-Measures ANOVA factor time, p < 0.01). Phosphorylation at serine 79 of ACC has an inhibitory effect on enzyme activity, indicating that ACC product, malonyl-CoA, a negative regulator of CPT1 activity, had lowest concentrations at this time point. CPT1 activity is then no longer inhibited and acyl-carnitine formation is high. According to these findings, the strong decrease in malonyl-CoA might be the rate-limiting step in increasing CPT1 activity in the periparturient dairy cow. Acyl-carnitines are then translocated by CACT across the inner mitochondrial membrane. Although mRNA amounts were not changed, CACT protein was expressed in significantly higher amounts at days +1 and +21 postpartum (Fig. 1.4). Thus, the next key process was the transfer of acyl-carnitines across the inner mitochondrial membrane. There, CPT2 releases carnitine from the acyl group and re-esterifies CoA to generate acyl-CoA ready to be utilized in the beta-oxidation in the mitochondrial matrix. While CPT2 mRNA expression was not affected, the amounts of protein showed a significant time effect with higher expression at days +1 and +21 postpartum (Fig. 1.5). According to the changes in expression and phosphorylation of key enzymes of the carnitine/acyl-carnitine shuttle system, the efficacy of this system might strongly contribute to the cow’s capacity to process NEFA for energy generation properly. After beta-oxidation, acetyl-CoA can be processed by the tricarboxylic acid cycle and respiratory chain. Only COX IV, an enzyme in the respiratory chain, was determined on protein level in this study; however expression was not affected by time (Fig. 1.6). The second fate for acetyl-CoA, when produced in excess, is ketone body generation. The mRNA expression of the key ketogenic enzyme HMGS2 was likewise not affected by time (Fig. 1.7). Expression of AMPK, an energy sensor, and extent of its phosphorylation was also not affected by onset of lactation (Supplementary Fig. S1). The latter components of energy metabolism need to be examined in further studies in dairy cows.

To sum up, mitochondrial fatty acid transfer through the carnitine/acyl-carnitine shuttle system was regulated at two major key points in dairy cows: firstly, the entry step of acyl to acyl-CoA mediated by ACSL1 and secondly, the transfer of acyl-carnitine into the inner matrix mediated by ACC (via malonyl CoA) and CACT. CPT1 expression is suggested to be constitutively high and its activity is regulated by the level of malonyl CoA, while CPT2 protein is slightly increased after parturition. Differences in expression levels of these key enzymes and also in enzyme activities (not yet determined) between cows explain, at least in part, the high inter-individual variation in dairy cows’ capacity to adapt to onset of lactation.

However, mitochondrial genotype may also play a role in metabolic adaptation to catabolic conditions. Thus, in trial 2, mtDNA haplotypes were identified and liver fat and glycogen content and plasma metabolites were associated with the haplotypes.

### Identification of mitochondrial genotypes (mtDNA haplotypes)

**Figure 2:**
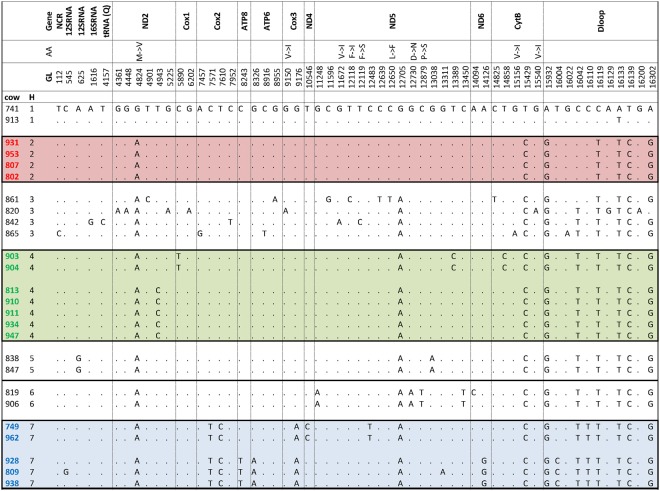
Sequence polymorphisms of hepatic mtDNA of Holstein dairy cows. About 97% of the whole mitochondrial genome of each individual cow was sequenced and aligned to the sequence of cow No. 741. For each nucleotide polymorphism, mtDNA region and alignment position are given. Identical nucleotides were indicated by dots; polymorphisms are given in one letter abbreviation of nucleotides. Genomic location (GL) was based on the BLAT analysis against *Bos taurus* genomic assembly (UMD3.1). Non-silent mutations are indicated in the line “AA”, amino acids were given in the one letter code (arrow between amino acids indicate direction of exchange). Haplotypes (H) separated by genetic distance analysis (see also Fig. 3) were numbered 1–7 (color codes of H 2, 4 and 7 according to Fig. 3). NCR: non coding region, tRNA (Q): transfer RNA (glutamine), ND2: NADH dehydrogenase subunit 2; Cox1: cytochrome c oxidase subunit I; ATP8: ATP synthase F0 subunit 8, ATP6: ATP synthase F0 subunit 6, Cox3: cytochrome c oxidase subunit III, ND4: NADH dehydrogenase subunit 4, ND5: NADH dehydrogenase subunit 5, ND6: NADH dehydrogenase subunit 6, CytB: cytochrome b, Dloop: displacement loop

**Figure 3:**
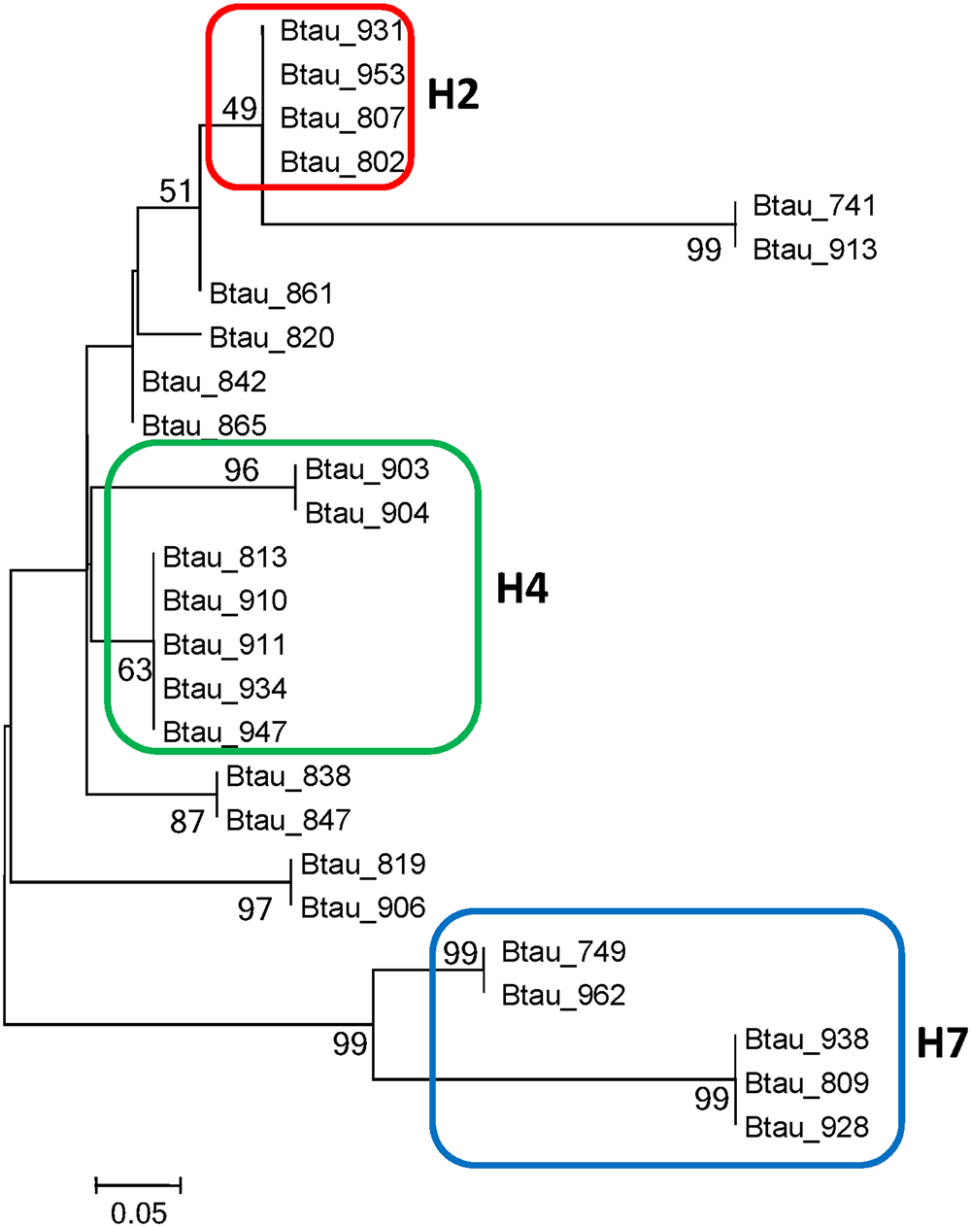
Maximum likelihood tree based on mtDNA sequences of Holstein dairy cows. Sequence information for over 97% of the hepatic mitochondrial genome of 26 cows (*Bos taurus*) was subjected to maximum likelihood tree reconstruction, based on the model of Kimura 2-parameter nucleotide genetic distances per informative site (n = 28). The cows belonging to 3 major haplotypes, H2, H4 and H7 are indicated in the red, green and blue-line boxes, respectively, with each haplotype name. Btau = *Bos Taurus*.

Nucleotide sequences of mtDNA covering 97% of the total mitochondrial genome were determined by PCR and Sanger sequencing using DNA isolated from liver biopsy samples of 26 healthy Holstein dairy cows. In total, 57 genomic locations of single nucleotide exchanges were found (Fig. 2), generating a low genetic diversity (π = 5.2 × 10^−4^) but high haplotype diversity (Hd = 0.93). Twelve mutations were identified in non-coding region and D loop region, 3 in ribosomal RNA, and 1 in transfer RNA, while the other 41 mutations were found in the region encoding 10 different genes. Within these, 10 mutations were nonsynonymous substitutions accompanied by exchanges of amino acids. Based on these mutations, we identified 15 different mtDNA sequence variants within the 26 cows. Taking the types of mutation (synonymous or not) and the results of the maximum likelihood tree analysis into account, these 15 sequence variants were assigned to 7 major mtDNA haplotypes (Figs 2 and 3). Three of these 7 haplotypes were found in at least 4 cows each, and 16 of the 26 cows belonged to one of these three haplotypes. Consequently, 3 haplotypes (H), H2 (n = 4), H4 (n = 7), and H7 (n = 5) were created based on similarity and used for the further analyses.

As mentioned above, D loop variations of mtDNA were often used for analysis of polymorphisms in cows and for classification of genotypes related to different maternal lineages^12,13^. Polymorphic nucleotides were identified at eight different positions in the D loop of bovine mtDNA^18^. Between cows of different Chinese breeds a total of 57 haplotypes could be identified by sequence analysis of mitochondrial D loop^19^. Some of the mtDNA mutations found in individual Holstein cows of this study across all haplotypes were also identified - beside others - in a Holstein research herd in North Carolina (C to T at position 8916; G to A at position 16200; G to A at position 16022^20,21^). Identified haplotype clusters (H2, H4, H7) were mainly based on sequence variations located in other parts of the mtDNA. These parts were identified to encode for COX 1, 2 and 3 subunits, ATP synthase subunits 6 and 8, cytochrome b and NADH dehydrogenase subunits 4, 5 and 6. Since these genes determine the OXPHOS capacity of mitochondria, mtDNA haplotypes based on polymorphisms in functional key genes might be closely associated with phenotypic characteristics of dairy cows’ metabolism and thereby with their capacity to adapt to onset of lactation. The role of silent mutations needs further examination.

### Matching phenotypic characteristics to mtDNA haplotypes

**Figure 4:**
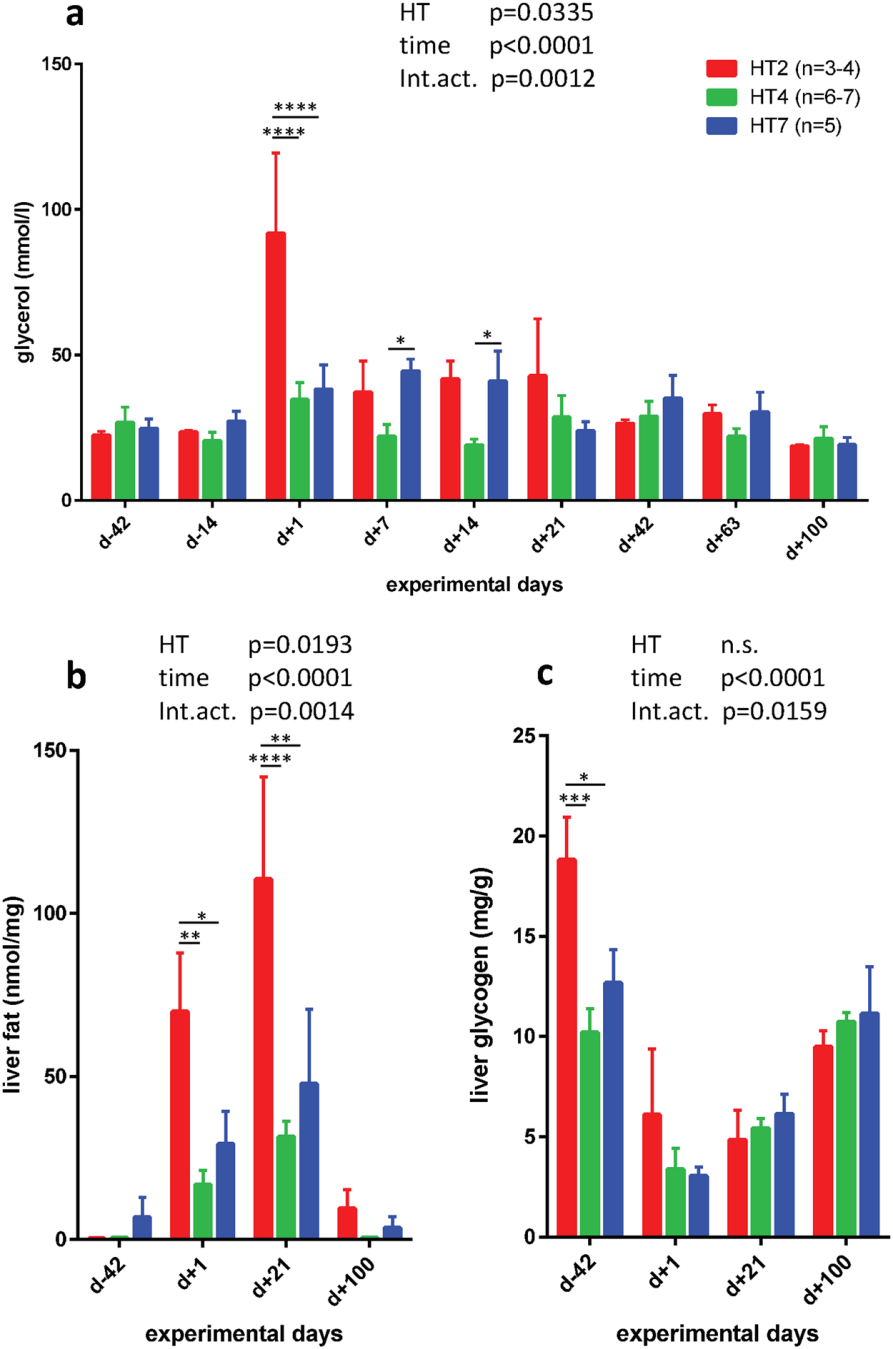
Matching energy metabolism-related characteristics to mtDNA haplotypes. (**a**) Serum glycerol, (**b**) liver fat and (**c**) glycogen concentrations and their association with mitochondrial haplotypes in periparturient dairy cows (means ± SEM). Data were analysed using Two Way ANOVA for factor haplotype (HT) and factor time and interactions (Int.act.) (see inserts), respectively, with Tukey’s multiple comparison posttest. Differences between mtDNA haplotypes at the same sampling day were indicated with *p < 0.05, **p < 0.01, ***p < 0.001 and ****p < 0.0001.

To match phenotypic characteristics to mtDNA haplotype, only the subset of 16 Holstein cows was used that could be allocated to haplotype H2, H4 and H7. These cows were phenotypically characterized in-depth previously, and those data were already published elsewhere^2,14–17^. Serum glycerol, beta-hydroxybutyrate (BHBA) and NEFA concentrations, liver fat and glycogen contents and serum acyl-carnitines were used to match the 16 individual phenotypes with identified mtDNA haplotypes. While NEFA and BHBA concentrations in serum were not associated with one of the identified haplotypes (data not shown), glycerol concentrations showed a clear association with mtDNA haplotype with an extremely high concentration at d + 1 postpartum in cows carrying mitochondrial haplotype H2 (Fig. 4a). Furthermore, liver fat concentration was significantly associated with mtDNA haplotype H2 expressing higher concentrations at d +1 and d +21 postpartum (Fig. 4b). Thus, cows carrying mtDNA haplotype H2 expressed signs of enhanced lipid mobilization compared to haplotypes H4 and H7, as indicated by higher glycerol concentrations in serum postpartum. Glycerol is a valid marker for rate of lipolysis in adipose tissues with increased concentrations at higher stimulation of hormone-sensitive lipase^22^. Obviously, as a consequence of higher lipid mobilization and NEFA release, NEFA were taken up in the liver efficiently, because no association of serum NEFA concentrations with higher glycerol values was observed in these haplotype H2 carriers. As indicated by higher liver fat content in haplotype H2 carriers at d +1 and d +21 postpartum compared to haplotypes H4 and H7, hepatic re-esterification of NEFA to triacylglycerides was the preferred pathway to utilize NEFA but not to oxidize them. Thus, cows with mtDNA haplotype H2 shifted fat from adipose tissues to the liver what may disturb hepatic metabolism and promote hepatic steatosis. In addition, these cows had higher glycogen stores in liver at d −42 prepartum (Fig. 4c), a store which is used postpartum for maintaining serum glucose. This supports glucose flux to the udder and serves as important intermediate to keep the tricarboxylic acid cycle running. The glycogen store was becoming depleted early postpartum, reaching similarly low levels in all haplotypes, and mtDNA haplotype H2 carriers did not restore their relatively large glycogen store, that was observed on d −42, during the monitored postpartum period (Fig. 4c).

**Figure 5:**
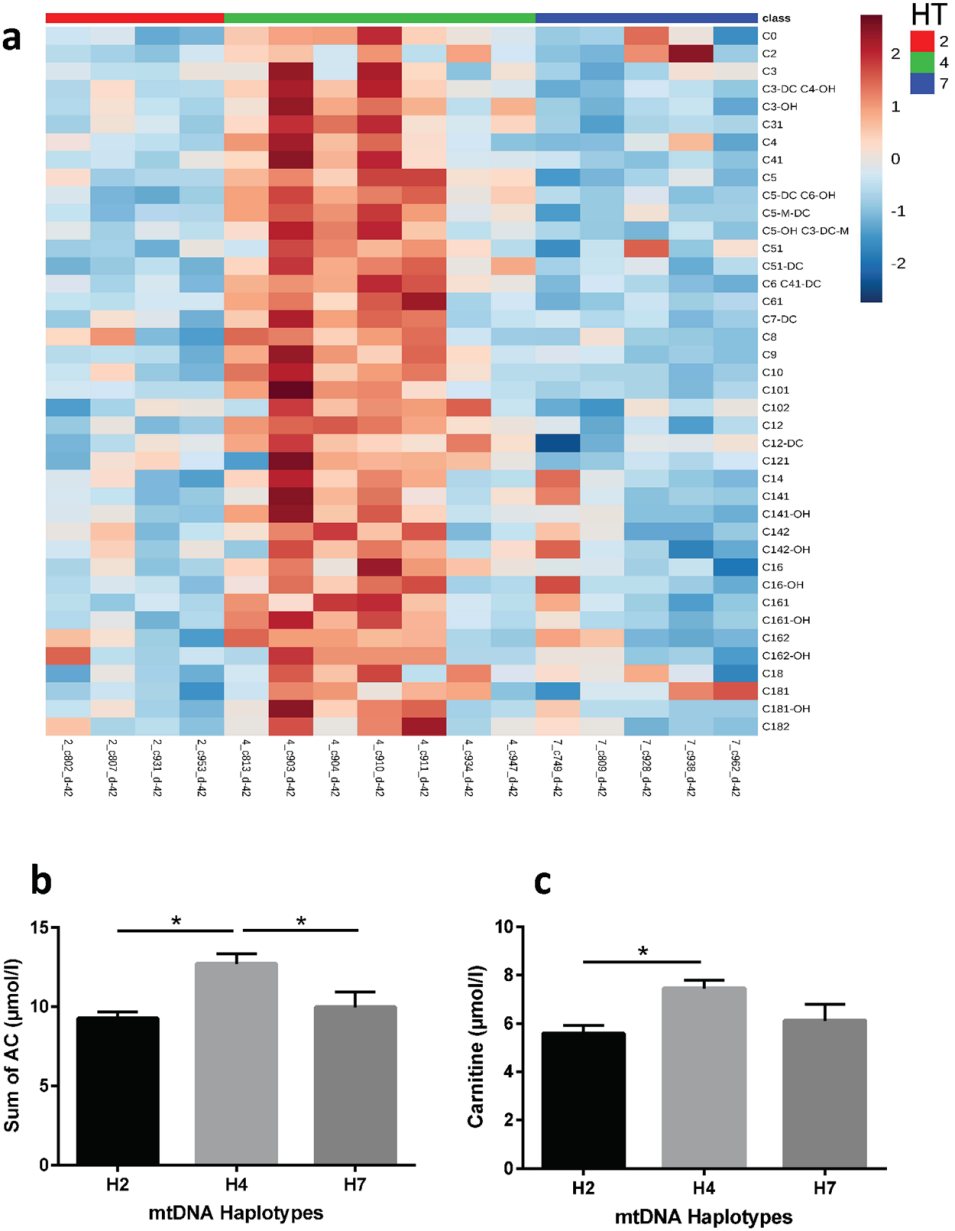
Acyl-carnitines (AC) in plasma and their association with mitochondrial (mt) DNA haplotypes in Holstein dairy cows. (**a**) Heatmap (http://www.metaboanalyst.ca/) of plasma AC at day −42 prepartum of haplotypes (class) 2, 4 and 7 showing individual cows, clustered to their respective haplotype. (**b**) Means of plasma AC sum (±SEM; p = 0.0122) and (**c**) means of carnitine (±SEM; p = 0.0411) in cows of the three mtDNA haplotypes at day −42 prepartum. Statistical differences were tested by One Way ANOVA with Tukey’s multiple comparison posttest. Significant differences between mtDNA haplotypes were indicated by *p < 0.05.

Using a metabolomics approach, acyl-carnitine concentrations were determined in serum of the 16 dairy cows of haplotype H2, H4 and H7^16^. These serum acyl-carnitines were mainly derived from liver energy metabolism^3^. Since acyl-carnitines result from the activities of hepatic ACSL1 (acyl-CoA formation) and CPT1 (acyl-carnitine formation), their concentration in serum was assessed as indicator for proper mitochondrial activity in general. Furthermore, their occurrence in serum was interpreted as protective response of mitochondria to avoid lipotoxic damage^3^. For d −42 prepartum, data were demonstrated as a heatmap (Fig. 5a) to visualize levels of acyl-carnitines within the individual cows of different haplotypes. On this day, clustering according to acyl-carnitines matched haplotypes the most (Supplementary Fig. S2). Sum of acyl-carnitines (Fig. 5b) and carnitine (Fig. 5c) concentrations were significantly higher in haplotype H4 compared to haplotype H2 at d −42 prepartum. However, H4 carrier cows expressed a high inter-individual variation. The association of acyl-carnitines with mtDNA haplotype was only observed prepartum but disappeared postpartum during onset of lactation (Supplementary Fig. S2). This indicated that plasma acyl-carnitine levels were affected by superposing factors under tensed metabolic conditions, reducing the influence of the mtDNA haplotype effect. The metabolic role of this particular pattern observed in serum carnitine and acyl-carnitine concentrations during the periparturient period and its regulation remain unclear and need further scientific evaluation.

Hypothetically, if high serum acyl-carnitines signal a proper mitochondrial activity and efficient oxidative ATP energy production, this might explain the significant lower lipolysis rate (as indicated by lower glycerol concentrations at d +7 and d +14 postpartum and the lowest liver fat content at d +1 and d +21 postpartum in haplotype H4 carriers (Fig. 4a,b). The lower concentrations of plasma acyl-carnitines coupled with the highest liver fat content in haplotype H2 supported the hypothesis, implying that this mtDNA haplotype was less able to utilize NEFA for oxidative ATP energy production due to a reduced capacity to form acyl-carnitines. In addition, this haplotype might also express a lower efficiency to release acyl-carnitines, thereby creating a negative feedback inhibition of NEFA activation by ACSL1 and acyl-carnitine formation by CPT1. Nevertheless, it should be noted that these considerations are based on the hepatic level. To assess efficiency on a more holistic level, future studies should extend the analysis of the fate of fatty acids to skeletal muscle and mammary gland in mtDNA haplotype characterized cows. The metabolic conditions of haplotype H7 with low acyl-carnitine concentrations and intermediate liver fat and glycogen content is unclear so far. Most likely, other factors such as nuclear-encoded gene products may modulate mitochondrial metabolism in specific haplotypes. Unfortunately, the expression data of mitochondrial energy metabolism generated in trial 1 were not obtained from a higher number of animals per haplotype group, because this examination was done before determination of mtDNA haplotypes in a selected set of liver biopsies.

In general, since the number of animals per haplotype was very low, all demonstrated results could only be used to generate new hypotheses about potential relationships between mtDNA haplotypes and metabolic phenotypes in Holstein dairy cows. However, there is strong evidence from this study, that important metabolic features such as liver fat content, lipolysis rate and molecular pathways involved in acyl-carnitine metabolism are related to certain mtDNA haplotypes. As a consequence, the maternal influence on metabolic health should be considered in breeding concepts in the future. For that, these findings must be extrapolated to and validated in a larger group of dairy cows in future studies in science and in the dairy field. Since mitochondrial functionality is an important physiological feature also for ageing in all living organisms with oxidative ATP generation, the relationship between mtDNA haplotype and metabolic phenotype may also have implications for human health, especially because of the high similarity of human and bovine mitochondrial genome^7^.

## Methods

### Animals and data sources

The experiment and sampling was conducted in 2013-2014 at the experimental station of the Institute of Animal Nutrition, Federal Research Institute of Animal Health (FLI) in Braunschweig, Germany, according to the European Community regulation concerning the protection of experimental animals and was approved by the Lower Saxony State Office for Consumer Protection and Food Safety (LAVES), Oldenburg, Germany (File number 3392 42502-04-13/1102). The cows used in this study were a subset of animals involved in a feeding trial to test the effect of energy density in the diet and niacin supplementation. Animals selected for the present paper were balanced across nutritional treatment groups; even though the diet was assumed to have no effect on the investigated parameters since production performance and various physiological measures remained unaffected by the applied diet as shown in our previous studies^14–17^. The animals were kept in a free stall housing system with free access to water. Except for the amount of protein and mRNA of mitochondrial proteins, liver glycogen and mtDNA haplotype identification presented in this study, all other data applied for the current analysis were previously published elsewhere^2,14–17^. Thus, methodological details were only given for the new data sets described in this study.

### Blood and liver biopsy sampling

The sampling procedure was described in detail previously^17^. Blood samples were obtained by jugular venepuncture at days −42 and −14 before the expected calving date (d − 42 and d − 14) and 1, 7, 14, 21, 42, 63, and 100 days postpartum (d + 1, d + 7, d + 14, d + 21, d + 42, d + 63, and d + 100, respectively). Liver biopsy samples were taken at d − 42, d + 1, d + 21, and d + 100 at the 10^th^ or 11^th^ intercostal space on the right side under ultrasonographic control after infiltration anaesthesia with 6 ml procaine using an automatic device for biopsy sampling and a commercial Tru-Cut biopsy needle (Bard Magnum, Tru-Cut 12 G needle, Bard Biopsy System, Tempe, AZ, USA).

### Total RNA isolation and real-time quantitative polymerase chain reaction (real-time PCR)

The methods of real-time PCR used in this study were described in detail elsewhere^17^. About 30 mg frozen liver biopsy sample was homogenized and total RNA was isolated using a commercial kit (RNeasy® Mini Kit, Qiagen, Venlo, Netherlands). The concentration and purity of isolated RNA was determined by measuring absorbance OD at 230, 260 and 280 nm (Biophotometer, Eppendorf AG, Hamburg, Germany). The integrity of RNA samples was controlled using RNA Integrity Number based on electrophorogram measured with an Agilent 2100 bioanalyzer and a commercial kit (RNA Nano Chips, Agilent Technologies, Santa Clara, CA, USA). One µg of total RNA was applied to reverse transcription performed using a commercial kit (iScript cDNA Synthesis Kit, Biorad Laboratories, Inc., Hercules, CA, USA) according to the manufacturer’s instructions with a final volume of 20 µl. Transcribed cDNA samples were diluted at 1:20 with nuclease-free water and stored at −20 °C for further analysis. No-template control and no-reverse-transcriptase controls were included in the assay. Quantitative real-time PCR was performed using the CFX96 Connect^TM^ Real-Time PCR Detection System (Biorad Laboratories, Inc., Hercules, CA, USA) and a commercial PCR master mix (SsoAdvanced™ Universal SYBR® Green Supermix, Biorad Laboratories, Inc., Hercules, CA, USA). The reaction mix consisted of PCR master mix, forward and reverse primer (500 nM each), 2 µl of the diluted cDNA samples (corresponding to 5 ng of RNA), and nuclease-free water with a final volume of 20 µl. The protocol of thermal cycling was 30 sec at 95 °C for polymerase activation, followed by 40 cycles of 10 sec at 95 °C and 15 sec at 60 °C for amplification and melt-curve analysis from 65 to 95 °C, 5 sec at 0.5 °C increments. The PCR assay was performed in triplicate for each sample. Each assay contained no-template controls and five dilution series of standards corresponding to 1.25–20 ng RNA and inter-plate controls. Primers were selected using “Primer 3”^23^. Specificity of the PCR products were tested by melt-curve analysis, agarose gel electrophoresis, and sequencing (Eurofins Genomics GmbH, Ebersberg, Germany). Investigated genes, primer sequence and results of melt-curve and sequence analysis of PCR products were shown in Supplementary Table S1A. Ribosomal protein L19 (RPL19) and ribosomal protein L32 (RPL32) were selected as the most stably expressed genes among the seven candidate genes by geNorm analysis and used as reference genes. The results of qPCR assay were analyzed using qBase Plus (Biogazelle NV, Zwijnaarde, Belgium). Normalized relative quantities of RNA for target genes were calculated from the threshold cycles (Cq) at relative fluorescence units of 50 using the delta-delta-Ct based method modified for the use of multiple reference genes for normalization^24^.

### Total protein isolation and immunoblot

The methods for total protein isolation and immunoblot were described in detail elsewhere^17^. About 45 mg of liver biopsy samples were weighed and homogenized in 800 µl of lysis buffer containing protease inhibitors (cOmplete, Mini, F.Hoffmann-La Roche Ltd., Basel, Switzerland) and phosphatase inhibitors (PhosStop; F. Hoffmann-La Roche Ltd.) with ceramic beads, followed by a centrifugation at 9000 g for 5 min at 4 °C. The protein concentration of supernatant was measured using the protein assay according to Bradford (Bradford Reagent, 5×, SERVA, Heidelberg, Germany). The protein extracts were stored in −80 °C until further analysis. Thirty µg of the extracts were applied to SDS-PAGE (Mini-Protein Tetra Cell, Bio-Rad Laboratories, Inc., Hercules, CA, USA) and transferred to a nitrocellulose membrane (Bio-Rad Laboratories, Inc., Hercules, CA, USA) using the semi-dry blotting system (Trans-Blot Turbo system, Bio-Rad laboratories, Inc., Hercules, CA, USA). Membranes were treated with blocking buffer (5% BSA) for one hour at room temperature, washed three times with washing buffer for 5 min each. Membranes were incubated with primary antibodies over night at 4 °C, followed by three wash cycles of 5 min and incubated with secondary antibodies for 1–2 h. Membranes were then washed three times for 5 min with washing buffer and once for 10 min with the washing buffer without Tween 20 and incubated with LumiGLO substrate (Kirkegaard & Perry Laboratories, Inc., Gaithersburg, MD, USA). The detection and recording chemiluminescence signals of membranes were performed using a Molecular Imager ChemiDoc XRS + System (Bio-Rad Laboratories, Inc., Hercules, CA, USA). Antibody conditions for each protein are presented in Supplementary Table S1B. The normalization for the equalities of loading for each signal was performed using beta-actin. Inter-membrane control samples were applied to all the membranes. All the primary antibodies were predicted to react with the bovine target proteins by the respective manufacturers. The specificity of detected signal for the target proteins was proved by the molecular weight and negative controls. Representative membrane images are shown in Supplementary Fig. S3.

### Glycogen concentration in the liver

The homogenate of liver sample prepared for protein extraction was used for glycogen assay. After the homogenization, 100 µl of the homogenates were mixed with 900 µl distilled water, heated at 99 °C for 10 min to deactivate enzymes and centrifuged at 18,000 g for 10 min at 4 °C. The supernatant was stored in −20 °C until further analysis. The glycogen concentration was measured colorimetrically (GENios Pro, Tecan Group Ltd., Männedorf, Switzerland) using a commercial kit (Glycogen Assay Kit, BioVision Inc., Milpitas, CA, USA).

### Isolation of total DNA from liver tissue, amplification of mitochondrial DNA, sequencing analysis

The total DNA was isolated from liver biopsy samples using a commercial kit (Wizard Genomic DNA Purification Kit, Promega, Manheim, Germany) according to the manufacturer’s instruction. The concentration and the purity of DNA were examined from absorbance at 260, 280, and 230 nm using the Nanodrop 1000 spectrophotometer (VWR International, Radnor, PA, USA). The samples were stored at −80 °C until further analysis. For the amplification of mitochondrial DNA, PCR was set up by adding 10–20 ng of the total DNA template, the reaction mix containing 500 nmol/l primer (Eurofins Genomics, Ebersberg, Germany), 200 µmol/l dNTPs (Thermo Fisher Scientific, Inc., Waltham, MA, USA), 0.02 U/µl Q5® High-Fidelity DNA Polymerase (New England Biolabs, Inc., Ipswich, MA, USA), PCR buffer with 2 mM MgCl_2_, and water with the final volume of 25 µl. The samples were incubated at 98 °C for 30 sec, 35 cycles of 95 °C for 10 sec, at 62 or 52 °C for 30 sec, 72 °C for 60 sec, and finally at 72 °C for 2 min (Master cycler Pro S, Eppendorf, Germany). The specific amplicon size of the PCR-products was confirmed by the 1% agarose gel (VWR International, Radnor, PA, USA) electrophoresis in TBE buffer. The PCR-products were purified using a commercial kit (PCR-Purification kit, Qiagen, Hilden, Germany) according to the manufacturer’s instruction. Purified PCR products were sent for Sanger-sequencing analysis service (GATC Biotech AG, Konstanz, Germany) using the two primers used for the PCR. Three primers were additionally used for sequencing analysis only. Information about the primer used for PCR and sequencing is presented in Supplementary Table S1C. PCR products were sequenced in two independent sequencing reactions, using the reverse and the forward DNA strand.

### Data analysis and statistics

#### Identification of haplotypes

DNA sequence assembly was performed using the software BioEdit^25^. The final sequences were applied for BLAST-like alignment tool (BLAT) offered by Ensembl (http://www.ensembl.org)^26^ against *Bos taurus* genomic assembly (UMD3.1) to determine the genomic location. The sequences of all the fragments were integrated into a sequence based on the genomic location and applied to further analysis. Multiple alignment analysis and genetic distance analysis by Maximum likelihood algorithm were conducted using MEGA version 7^27^. Bootstrapping was performed with 1000 replicates.

#### Clustering of metabolites

Heatmaps to illustrate relative serum concentrations of acyl-carnitines were created by using the software MetaboAnalyst (http://www.metaboanalyst.ca/). Clustering of samples was performed according to Euclidean distance measure and average cluster algorithm, all features were autoscaled.

#### Gene and protein expression and metabolite concentrations in plasma

The effect of time on gene and protein expression in liver was tested by One Way Repeated-Measures (RM) ANOVA and associations of metabolic variables with haplotypes were tested by Two Way RM ANOVA with Tukey’s multiple comparison posttest. Statistical differences between haplotypes were tested by One Way ANOVA with Tukey’s multiple comparison posttest. All analyses were done using GraphPad Prism (version 7.02; https://www.graphpad.com/scientific-software/prism/). Level of significance was set at p ≤ 0.05.

## Electronic supplementary material

Supplementary Information

## Data Availability

The mitochondrial DNA sequence data analysed during the current study are available in the GenBank under the accession numbers MK028725 to MK028750. All other datasets generated during or analysed during the current study are available from the corresponding author on reasonable request.
