# Supplementary material for: Inter-individual variation in adaptive capacity at onset of lactation: Linking metabolic phenotype with mitochondrial DNA haplotype in Holstein dairy cows
Source: Sci Rep. 2018 Oct 18;8:15439. doi: 10.1038/s41598-018-33853-6 (PMC6193931; doi:10.1038/s41598-018-33853-6)

Inter-individual variation in adaptive capacity at onset of lactation: Linking metabolic phenotype with mitochondrial DNA haplotype in Holstein dairy cows

Asako Kinoshita[1+], Ákos Kenéz[1+#], Martin Hasselmann[1], Sven Dänicke[2], Korinna Huber[1*]

[1]Institute of Animal Science, Faculty of Agricultural Sciences, University of Hohenheim, Stuttgart, 70599, Germany
[2]Institute of Animal Nutrition, Friedrich-Loeffler-Institute, Braunschweig, 38116, Germany

*Corresponding author: Korinna Huber (korinna.huber@uni-hohenheim.de)
[+]These authors contributed equally to this work
[#]Current address: College of Veterinary Medicine and Life Sciences, City University of Hong Kong, Kowloon, Hong Kong SAR

Supplementary Information

Supplementary Table S1A
Supplementary Table S1B
Supplementary Table S1C
Supplementary Figure S1
Supplementary Figure S2
Supplementary Figure S3

Supplementary Table S1A

Name and GenBank accession number of investigated genes by real-time qPCR

Gene symbol	Gene name	Accession number[2]	Metabolic pathway
ACCA	acetyl-coenzyme A carboxylase alpha	NM_174224	Synthesis of malonyl-CoA in fatty acid synthesis pathway
CPT1A	carnitine palmitoyltransferase 1A	NM_001304989.1	Transport of fatty acid into mitochondria through outer mitochondrial membrane
CPT2	carnitine palmitoyltransferase 2	NM_001045889	Transport of fatty acid into mitochondria through inner mitochondrial membrane
HMGS2	3-hydroxy-3-methylglutaryl-coenzyme A synthase	NM_001045883	Synthesis of 3-hydroxy-3-methylglutaryl-CoA in ketone body synthesis pathway
ACSL1	acyl-CoA synthetase long-chain family member 1	NM_001076085	Activation of fatty acid
CACT (SLC25A20)	solute carrier family 25 (carnitine/acylcarnitine translocase), member 20	NM_001077936.2	Transport of carnitines through inner mitochondrial membrane
RPL19[1]	60S ribosomal protein L19	NM_001040516	Ribosomal protein
RPL32[1]	60S ribosomal protein L32	NM_001034783	Ribosomal protein

[1]The assay information was described previously (Kinoshita et al., 2016)
[2]GenBank accession number for the template sequences

Assay information of genes measured by real-time qPCR

Gene symbol[1]	Primer Sequence[2]		Size[3]	Tm[4]
	Forward	Reverse		
ACCA	ATCCGACGCCTTACTTTCCT	TTCTCATCCGGTTCAGCTCT	193	82
CPT1A	CCTATTTTGGACACGGGAAA	TCAAACCACCTGTCGAAACA	172	86
CPT2	AGGCTGCCTATTCCCAAACT	GGACTTTGGATCAGGGTTGA	279	82.5
HMGS2	GGCCTTTCACTCTCGATGAT	TTCCAGCTTTAGTCCCCTGA	170	84.5
ACSL1	GAACTACAGGCAACCCCAAA	GGGCCTTGAGATCATCCATA	181	82.5
CACT (SLC25A20)	AGAGGGCATCAGGGGTCTAT	TGCAAATATCTGGGGGTAGC	151	85.5

[1]See Table 6 for the name of the gene for each gene symbol
[2]The exon-exon junctions were underlined
[2]The size of PCR products (bases)
[3]Melting temperatures of PCR products

Sequences of PCR-products

Gene symbo[l]	Sequence results
ACCA	TACAGTCACTATGAAGTGGATCAGAGATTTCATAGAGAATTTCCTAAATTTTTCACGTTCCGA GCAAGGGATAAGTTTGAGGAAGATCGTATCTATCGTCACCTGGAGCCTGCCCTAGCTTTCCAG TTAGAGCTGAACCGGATGAGAAAACTGAA
CPT1A	GAAGCAGCGTTCTTCGTGACGTTAGACGAAACCGAGCAGGGATACAGGGAGGAGGACCCGG AAACGTCGATGGACAGCTACGCCAAGTCCCTGCTGCATGGCAGGTGTTTCGACAGGTGGTTT GAATGGCTGCCT
CPT2	AGGCTGCCTATTCCCAAACTTGAAGACACCATTAGAAGATACCTCAGTGCACAGAAGCCTCT GTTGGATGACAGCCAGTTCAGGAAAACAGAACAGTTGTGTAAGAGTTTTGAAACTGGAATTG GAAAAGAACTGCATGAGCAGCTGGTCACTCAGGACAAGCAGAATAAACATACAAGCTACATT TCAGGCCCCTGGTTTGATATGTATTTAACTGCTCGAGATCCTGTTGTCCTGAACTTTAATCCGT TTATCTCATTCAACCCTGATCCAAAGTCCA
HMGS2	TTCACGCCTTTCTGCAGTTAGTCCAGAAATCCCTGGCCCGCCTGATGTTCAATGACTTCCTGTT GGCCAGTGGTGACACACAGACTGGCATCTACAAGGGCTTGGAGGCCTTCAGGGGACTAAAGC TGGAAAAGAACGCA

ACSL1	TGTGTAGTGAGCGATTGTTCAGCATTTGTGAAATGACAGAGAACACGTTCATTCCTACCTCAGATGACACTTTGATCTCTTTCTTGCCTCTGGCCCATATGTTTGAGAGAGTTGTAGAGTGTGTGATGCTCTGTCATGGAGCTAAAATAGGATTTTTCCAAGGAGATATCAGGCTGCTTATGGATGATCTCAAGGCCAA
CACT	AGGGCATCAGGGGTCTATATCGGGGCATGGCTGCCCCCATCGTCGGGGTCACCCCCATGTTTGCTGTCTGCTTCTTTGGGTTTGGTTTGGGGAAGAAACTGCAACAGAAACACCCAGAGGATGTGCTCAGCTACCCCCAGATATTTGCAA

[1]See Table 6 for the name of the gene for each gene symbol

Validation of PCR product sequences by BLAT search

Gene symbol[1]	Chromosome	Genomic Location	Overlapping Gene[1]	Length	E	%ID
ACCA	19	13852666-13852739	ACCA	74	8.30E-35	100.00
ACCA	19	13853790-13853831	ACCA	42	1.00E-15	100.00
ACCA	19	13859953-13859983	ACCA	31	4.70E-06	96.77
CPT1A	29	46841700-46841816	CPT1A	117	1.10E-60	100.00
CPT2	3	93612340-93612446	CPT2	107	1.10E-53	100.00
CPT2	3	93604196-93604284	CPT2	89	2.40E-43	100.00
CPT2	3	93614497-93614579	CPT2	83	4.40E-40	100.00
HMGCS2	3	23655814-23655928	HMGCS2	115	4.10E-56	99.13
ACSL1	27	14239367-14239444	ACSL1	78	6.10E-37	100.00
ACSL1	27	14237021-14237098	ACSL1	78	6.10E-37	100.00
ACSL1	27	14241820-14241859	ACSL1	40	2.20E-11	97.50
OCSN2	7	23331082-23331193	SLC22A5	112	9.40E-58	100.00
OCSN2	7	23332329-23332414	SLC22A5	86	2.00E-42	100.00
CACT	22	51630101-51630230	SLC25A20	130	2.60E-68	100.00
PPARA	5	117151604-117151699	PPARA	96	3.20E-45	98.96
PPARA	5	117195075-117195132	PPARA	58	9.20E-26	100.00

Results of BLAT search (www.ensembl.org) for sequences of PCR products of the investigated genes compared to cow genomic sequence assembly UMD3.1

E: Probability that the alignment between the query (input) sequence and subject (hit) sequence is due to chance, Length: the full length of the alignment, including all gaps in either the query or the alignment, ID: Percentage of the aligned query sequence which is identical to the subject sequence.

[1]See Table 6 for the name of the gene for each gene symbol

Supplementary Table S1B

Assay condition used for immunoblot analysis

Gene symbol	Primary antibody		Secondary antibody	
	Name	Condition	Name	Condition
ACC	ACC Cell Signaling	1:1000, ON, +4 ºC	Anti-rabbit HRP	1:2500, 1 h, RT
pACC	pACC Cell Signaling	1:1000, ON, +4 ºC	Anti-rabbit HRP	1:2500, 1 h, RT
AMPK	AMPKa Cell Signaling	1:800, ON, +4 ºC	Anti-rabbit HRP	1:2500, 1 h, RT
pAMPK	pAMPK Cell Signaling	1:1500, ON, +4 ºC	Anti-rabbit HRP	1:2500, 1 h, RT
CPT1A	CPT1A Novus Biologicals	1:500, ON, +4 ºC	Anti-rabbit HRP	1:2500, 1 h, RT
CPT2	CPT2 Novus Biologicals	1:1000, ON, +4 ºC	Anti-goat HRP	1:100000, 1h, RT
CACT	CACT Novus Biologicals	1:1000 ON, +4 ºC	Anti-rabbit HRP	1:2000, 1 h, RT
COX IV	COX-4 Cell Signaling	1:1500, ON, +4 ºC	Anti-rabbit HRP	1:2500, 1 h, RT

ON = overnight incubation (12 to 14 h), RT room temperature

Supplementary Table S1C

Primer information for sequencing analysis of mitochondrial genome

No	Primer Sequence		Position		Ann. temp	genes	Size
	Forward (for)	Reverse (rev)	for	rev			
1	CTGGACTTAACTGCATCTTGAGC	GTTTTGTTTTATTCTATCTTGGTT	128 - 150	1489 - 1512	53	16S, 12S	1385
2	GCACTAGCTACATGAGAGGAGACA	TTGGAAGTAAGAGACAGTAAAACC	1318 - 1341	2441 - 2464	64	12S, 16S	1146
3	AATGGCCGCACGAGGGTTTTA	ATGGAGCTCGGTTTGTTTCTGC	2427 - 2447	3671 - 3692	64	16S, ND1	1265
4	ATACGCACTAATCGGAGCCCTACG	GGATGCCCTGTGTTACTTCTGG	3478 - 3501	4611 - 4632	64	ND1, ND2	1154
5	ATAGCCCCATTTCACTTC	CGTTGTAGATTTCTCGTC	4587 - 4604	5834 - 5852	53	ND2, CoxI	1265
6	TTGGGCCGGTATAGTAGGAACAGC	TCGTCGAGGCATGCCAGATAGTC	5781 - 5758	6981 - 7003	64	CoxI	1222
7	CGACGATACTCCGACTA	TTTTATAATATTGACGCAGAT	6998 - 7015	8039 - 8059	53	CoxI, CoxII	1061
8	GTCCAGCTTATATTACGGTCAA	TAGGCCAATTATTAGCAGGGTCAT	7936 - 7958	9087 - 9110	64	CoxII, ATP8/6, CoxIII	1174
9	CTAATCGGAGGAGCTACACTTG	ATTTATTATTCTTTTTCGGACTA	8806 - 8827	9806 - 9828	53	ATP8/6, CoxIII	1022
10	TTATCACCATCACATTAGGAGTCT	TTTTCGGGTTAGGTTTTCTTTTGA	9460 - 9483	10781 - 10804	64	CoxIII , ND3, ND4	1344
11	CAGCCCTAACAATCCTCAACTCAC	TGGATAAGGATGGCTACGATAACG	10369 -10392	11416 - 11439	64	ND3, ND4	1070
12	GTAAGCCACATAGCACTCG	TTTGTTAATATTGGGGTCTG	11399 -11417	12437 - 12546	53	ND4, ND5	1147
13	GGAAAATATACCTACCACAT	CTATTATAAGGCCCAGTTGAC	11777 - 11796	13031 - 13051	64	ND4, ND5	1274
14	ACAATAGTGGTAGCAGGTA	TTGTTGATGGAGTTCTTTATGGTC	12859 -12877	14129 - 14152	64	ND5, ND6	1293
15	CTCCATCAACAAGCCAGTA	TGTGTAGTAGGGGGATTAGAGCA	14141 - 14159	15416 - 15438	64	ND6, CYTB	1297
16	CGAGGCTTATATTACGGGTCTTAC	GCGGGTTGCTGGTTTCAC	14811 -14834	16193 - 16193	64	CYTB Dloop	1382
17	AACACCACTAGCTAACATAACACG	GCCTTGCTTTGGGTTAAG	15898 - 15921	392-410	59.4	Dloop	1324

No. 1-16: GenBank accession number V00654. All the primers were used also for sequencing. ND1, ND2, ND3, ND4, ND5, and ND6: NADH dehydrogenase subunit 1, 2, 3, 4, 5, and 6, CoxI, CoxII, and CoxIII: cytochrome c oxidase subunit I, II, and III, ATP8: ATP synthase F0 subunit 8, ATP6: ATP synthase F0 subunit 6, CytB: cytochrome b, Dloop: displacement loop; AnnTemp= Annealing temperature

Primers used for sequencing analysis only

No.	Primer Sequence	direction	position
1	TCCTGTGACCATTGACTG	reverse	179 - 196
2	CGATAAACCTCACCAATTC	forward	1002 1020
3	AGGGATTGCGGCTTTATAC	reverse	14000-14018

Supplementary Figure S1
AMPK protein expression and phosphorylation

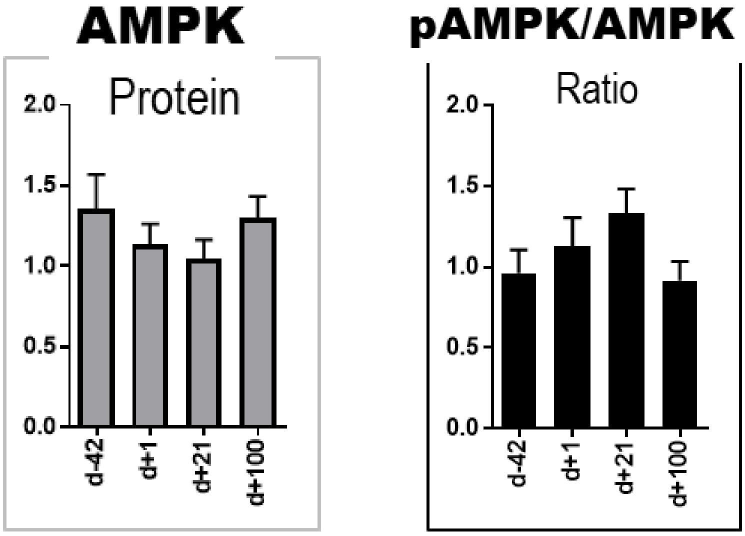

Supplementary Fig. S1. AMP activated protein kinase protein expression (AMPK) and protein phosphorylation (pAMPK; Ser485) in liver biopsy samples of Holstein cows (n=21), detected by Western blotting. Bar charts show expression and extent of phosphorylation (means±SEM) on different samplig days: day -42 prepartum, +1, +21 and +100 postpartum. One Way Repeated-Measures ANOVA showed no significant time effect: AMPK p=0.25, pAMPK p=0.17.

Supplementary Figure S2

Plasma carnitine and acylcarnitine concentrations

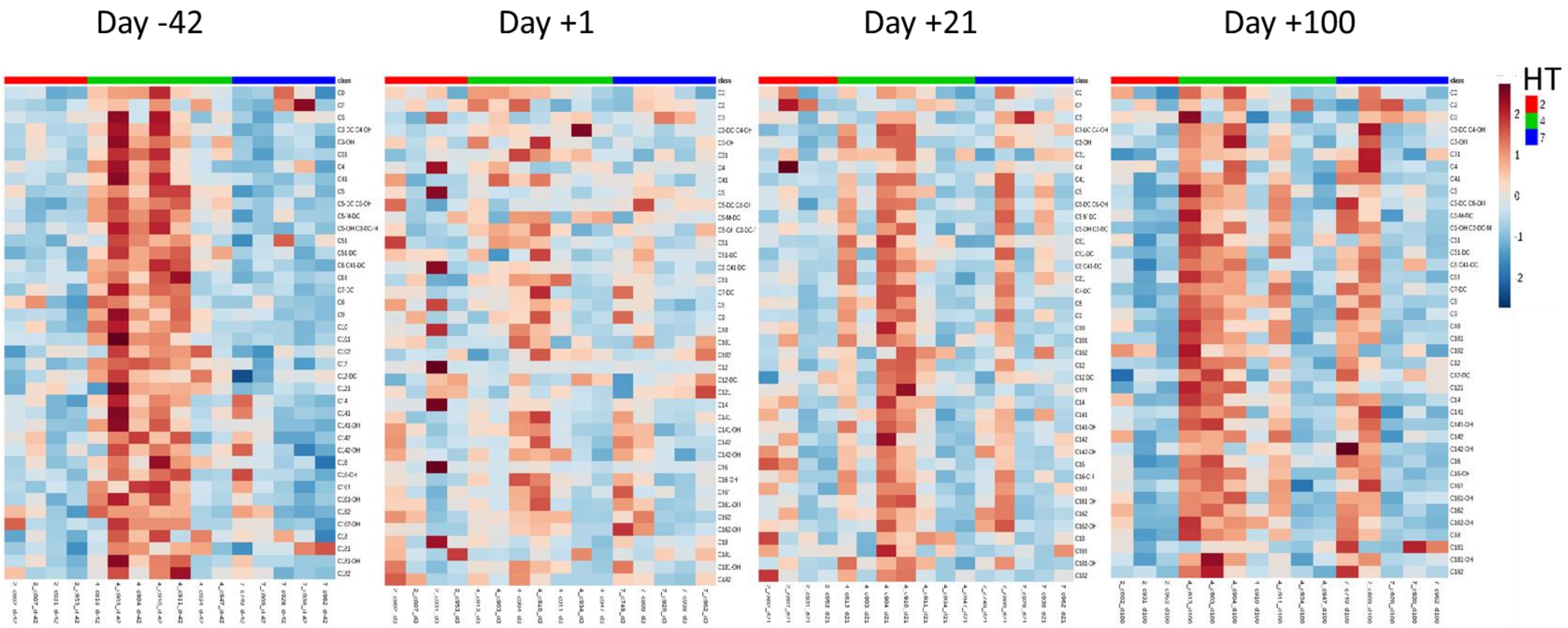

Supplementary Fig. S2. Visualization by heatmap (http://www.metaboanalyst.ca/) of carnitine (C0) and acyl-carnitine (AC) levels in plasma of periparturient dairy cows clustered by their mitochondrial DNA haplotype (HT) 2 (red), 4 (green) and 7 (blue). A clear association with the mtDNA haplotype was only observed at day -42; during the postpartum period the association was diminished. At day +100, four of the haplotype 4 carrier cows reached carnitine and AC levels similar to -42 day values; furthermore, in haplotype 7 carrier cows two individuals managed to have comparable high levels as haplotype 4 carriers.

Supplementary Figure S3

Representative Western blot membrane images of target proteins associated with energy metabolism in liver biopsy samples of Holstein cows.

Acetyl CoA Carboxylase (ACC; Cell Signaling #3662)

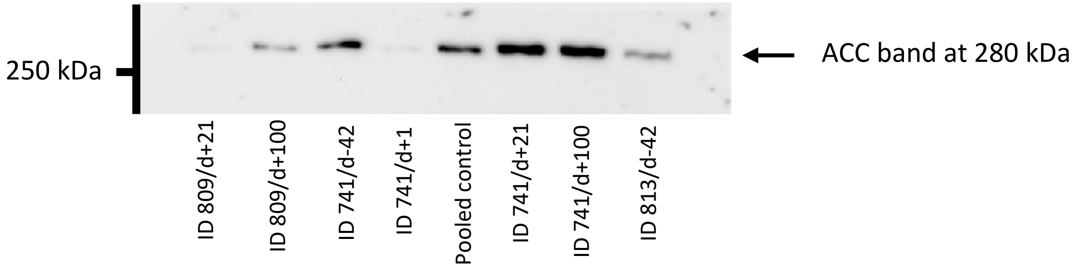

Phosphorylated Acetyl CoA Carboxylase (pACC; Ser79; Cell Signaling #3661)

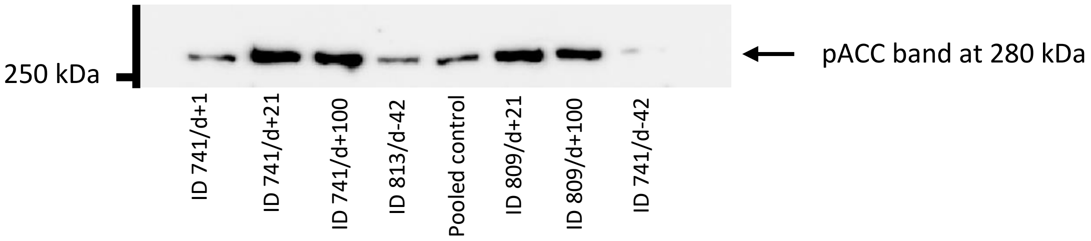

Carnitine palmytoiltransferase-1 (CPT1; Novus Biologicals NBP1-85473)

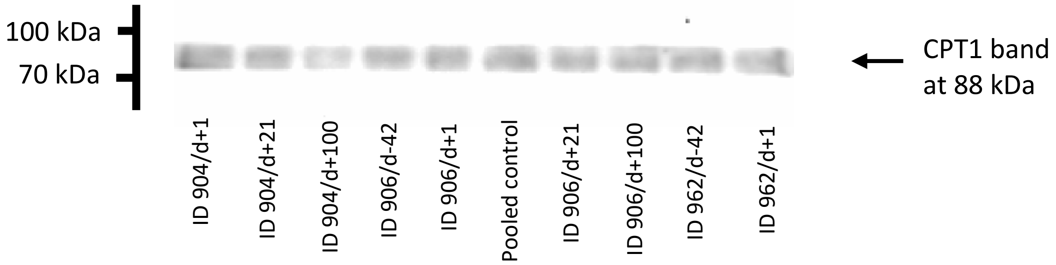

Carnitine-acylcarnitine translocase (CACT; Novus Biologicals NBP1-86689)

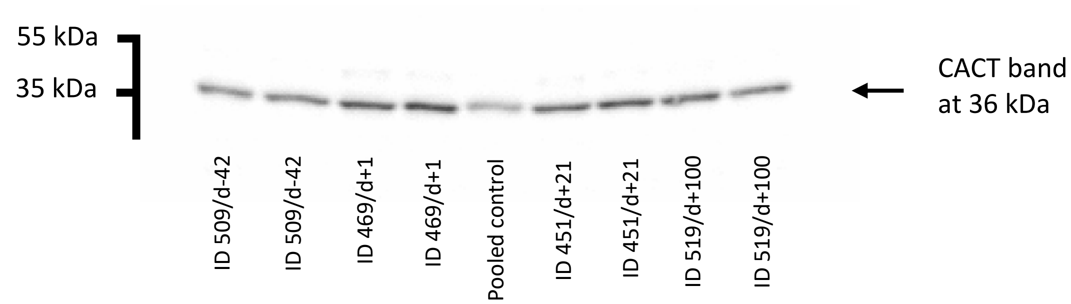

Carnitine palmytoiltransferase-2 (CPT2; Novus Biologicals NBP1-51993)

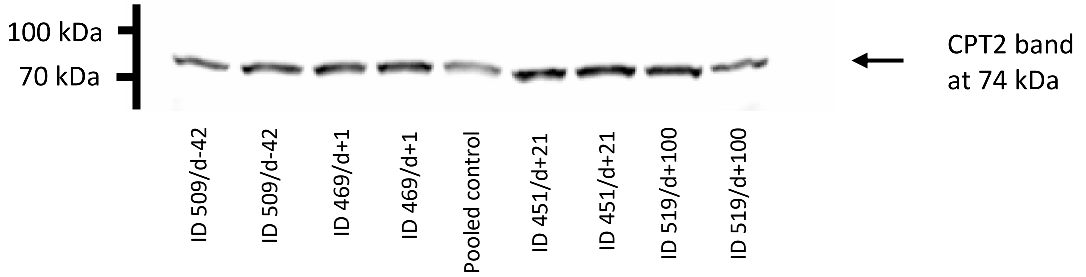

Cytochrome c oxidase IV (COX IV; Cell Signaling #4850)

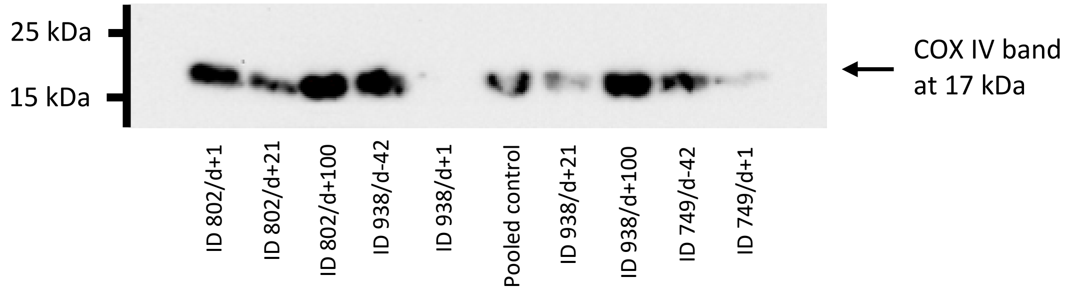

AMP activated protein kinase (AMPK; Cell Signaling #2532)

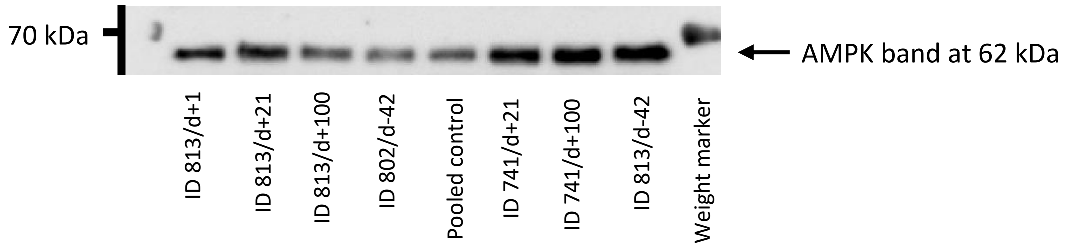

Phosphorylated AMP activated protein kinase (pAMPK; Ser485; Cell Signaling #4184)

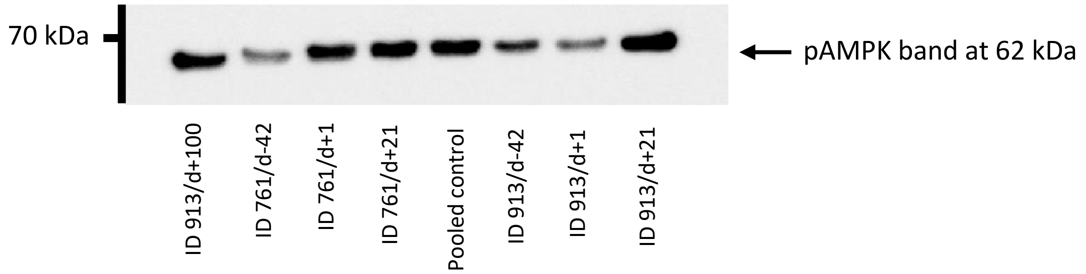

Beta-actin (Sigma-Aldrich A5316)

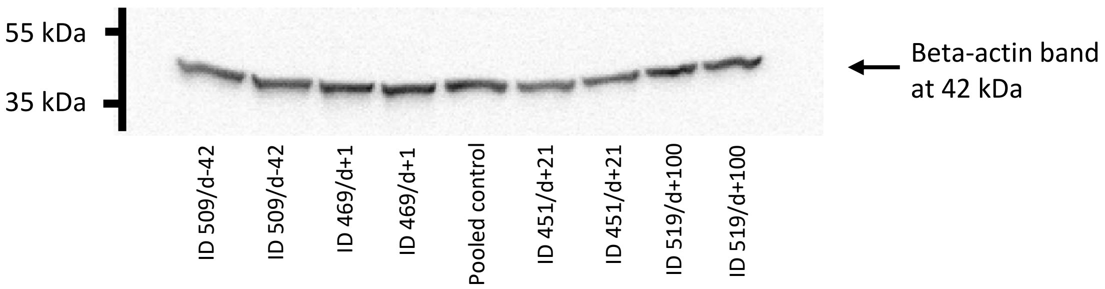

Supplement: Supplementary file 1 — Supplementary Information [file 41598_2018_33853_MOESM1_ESM.pdf]
